# Quality of Response in Acute Myeloid Leukemia: The Role of Minimal Residual Disease

**DOI:** 10.3390/cancers11101417

**Published:** 2019-09-23

**Authors:** Luca Maurillo, Renato Bassan, Nicola Cascavilla, Fabio Ciceri

**Affiliations:** 1 Hematology Unit, Department of Biomedicine and Prevention, Fondazione Policlinico Tor Vergata, Hospital, 00133 Rome, Italy; 2 Hematology Unit, dell’Angelo Hospital and Santissimi Giovanni and Paolo Hospital, 30174 Mestre and Venice, Italy; Renato.Bassan@aulss3.veneto.it; 3 Hematology Unit, Onco-Hematology Department, Fondazione IRCCS Casa Sollievo della Sofferenza, 71013 San Giovanni Rotondo (FG), Italy; n.cascavilla@operapadrepio.it; 4 Hematology and Bone Marrow Transplantation Unit, IRCCS S. Raffaele Scientific Institution, 20132 Milan, Italy; ciceri.fabio@hsr.it

**Keywords:** Acute myeloid leukemia, minimal residual disease, tailored therapy

## Abstract

In the acute myeloid leukemia (AML) setting, research has extensively investigated the existence and relevance of molecular biomarkers, in order to better tailor therapy with newly developed agents and hence improve outcomes and/or save the patient from poorly effective therapies. In particular, in patients with AML, residual disease after therapy does reflect the sum of the contributions of all factors associated with diagnosis and post-diagnosis resistance. The evaluation of minimal/measurable residual disease (MRD) can be considered as a key tool to guide patient’s management and a promising endpoint for clinical trials. In this narrative review, we discuss MRD evaluation as biomarker for tailored therapy in AML patients; we briefly report current evidence on the use of MRD in clinical practice, and comment on the potential ability of MRD in the assessment of the efficacy of new molecules.

## 1. Introduction

By definition, biomarkers are biological variables associated with the outcome of a given disease. Independent of the treatment received, a biomarker is defined as prognostic if it informs about clinical outcome (e.g., disease recurrence, disease progression, death), and it is defined as predictive if the treatment effect (experimental versus control) differs between biomarker-positive and biomarker-negative patients. Biomarkers can be either identified at diagnosis (e.g., genetic or molecular alterations; prognostic biomarkers), or during/after the administration of a given therapy to evaluate the quality of the obtained response (predictive biomarkers) [1]. Preferentially, biomarkers should be identified within the context of a clinical trial [1].

In acute myeloid leukemia (AML), multiple biomarkers have extensively been studied for their potential to predict outcomes with the goal to guide patients to tailored therapies with novel agents and to reduce chemotherapy-related toxicities [2,3]. In this context, a recent study by Gerstung et al., analyzed genomic and clinical datasets of 1540 patients by using multistage statistical models. In their study, the authors found that tailored treatment approaches led to a reduction in the number of hematopoietic stem cell transplants by 20–25% while maintaining overall survival (OS) rates [2]. 

In patients with AML who achieve a complete remission (CR), the minimal/measurable residual disease (MRD) is a well-recognized risk factor for relapse, which can be considered a broad predictive biomarker useful to guide the patient’s management and a potential surrogate endpoint for relapse-free survival (RFS) in clinical trials [4,5].

In this review, we discuss MRD evaluation as a biomarker for tailored therapy in AML patients and describe the current evidence on the use of MRD assays in clinical practice. We further comment on the potential ability of MRD technologies in the assessment of the antileukemic activity of novel agents.

## 2. Measurement of MRD: The Essential Tool for Precision Medicine

Increasing evidence suggests that morphological CR alone should no longer be considered as the only criterion to evaluate response to treatment [6]. In fact, detection of blast cells by light microscopy is hampered by limited sensitivity and inter-observer variability. On the other hand, the possibility of defining persistent disease (MRD) far below the morphological level of 5% blast cells is changing the landscape of response to initial treatment. For instance, the multicenter AML02 study clearly demonstrated that morphological assessment of treatment response has limited value compared with multiparameter flow cytometry (MFC), and that MRD positivity after induction was predictive of lower event-free survival and higher relapse rate (*p* < 0.001), thus providing strong prognostic information, regardless of the morphologic results [7]. Overall, different studies have demonstrated that, regardless of the technique employed, MRD persistence in a condition of morphological CR confers a negative prognosis that is comparable to that associated with morphological persisting leukemia [7,8]. 

Of note, the latest version of the recommendations issued by the European LeukemiaNet (ELN) points out that response may be categorized according to MRD status (Table 1). However, definite evidence of a specific intervention that may reduce the relapse risk in AML patients with a positive MRD test is lacking and represents an issue to be tested in prospective trials [9]. Therefore, at present, there is considerable interest in developing techniques to detect and/or quantify MRD, in order to estimate the likelihood and timing of relapse [9].

In line with this principle, an ideal MRD test would precisely quantify leukemia cells that are biologically able and likely to cause a leukemic relapse within a defined interval [9]. However, since AML is genetically heterogeneous, there is currently no uniform approach to detecting such cells. In addition, standardization and harmonization of assay platforms, as well as timing of assessment and result reporting are required to pave the way for MRD to become a more widely used surrogate endpoint for survival in clinical studies [10,11,12]. This harmonization will likely lead to improved assessment of the individual risk and may allow MRD to become more widely used as a surrogate endpoint for survival, in studies investigating new drugs, hopefully prompting faster drug approval in the AML setting [9].

## 3. MRD-Driven Strategy in Clinical Practice: Current Evidence

Definitive inclusion of MRD in the decision-making process requires that its benefit as a biomarker should be proven not only in retrospective cohorts but also in prospective controlled studies. Irrespective of the techniques used, several retrospective reports confirmed the prognostic role of MRD assessment. In the following paragraph, we briefly review current clinical evidence on the two most widely adopted methods to measure MRD, namely molecular biology-based approaches (e.g., real-time quantitative PCR (RT-qPCR)) and MFC.

### 3.1. Molecular Biology-Based Approaches

RT-qPCR allows MRD detection in cases with chimeric fusion genes generated by balanced chromosomal rearrangements. RT-qPCR is highly reproducible between laboratories, turnaround time is rapid, and risk of contamination with exogenous DNA (false-positive results) is substantially reduced. Optimized RT-qPCR assays are more sensitive than MFC, with a detection range of 10^−4^ to 10^−6^. 

A major drawback of RT-qPCR is its applicability only to those patients with molecular targets that are specific and stable over the treatment course, accounting for nearly 50% of young AML cases, and the possible contamination by non-viable cells carrying the same molecular target. Common targets of RT-qPCR are fusion genes generated by balanced chromosomal rearrangements (e.g., *PML-RARa* t(15;17), *RUNX1-RUNX1T1* t(8;21), *CBFB-MYH11* (inv(16) t(16;16), t(11q23) *MLL* fusions, *DEK-CAN(NUP214)* t(6;9)), insertions/duplications (e.g., *NPM1*, *FLT3-ITD*, *MLL-PTD*), point mutations (e.g., *CEBPA*, *IDH1/2*, *KIT*, *RAS*, etc.), gene overexpression (e.g., Wilms tumor-1 (WT-1)). Among these targets, *PML–RARa*, *NPM1*, *RUNX1–RUNX1T1*, and *CBFB–MYH11* have received an extensive clinical validation making them technical platforms suitable for application in clinical practice. 

Digital droplet PCR (ddPCR) is a newer high-throughput technology that can be used to directly quantify and clonally amplify nucleic acids and allows a more reliable collection and sensitive measurement of nucleic acid amounts with no need of a reference standard curve [13]. RNA or DNA molecules are fractionated into thousands of droplets, where each PCR amplification of the target gene occurs [14]. 

Despite the higher sensitivity compared with traditional RQ-PCR (up to tenfold) and precision, the major pitfall of ddPCR is that for each mutation a specific assay needs to be developed. As it is time consuming and costly, this assay is especially suitable for sensitive detection of recurrent mutations, such as *NPM1*, *IDH1*, and *IDH2* [14,15]. On the other hand, RQ-PCR actually still performs more than adequately for MRD detection in AML patients, is less expensive and is performed in many clinical laboratories already qualified for this molecular analysis [15]. 

Next-generation sequencing (NGS) technologies can be used to evaluate a few genes or an entire genome and provide the opportunity to study large number of somatic mutations in one single experiment [10]. This feature appears to be particularly useful in AML, where the wide intra-clonal heterogeneity often makes the leukemic clone a moving target. However, it should be taken into account that some persisting mutations, such as *DNMT3A*, *ASXL1*, and *TET2*, known to be present in clonal hematopoiesis (CHIP), actually do not have a prognostic role and that, currently, the sensitivity level is set at approximately 1%, which cannot compete with other MRD measurements techniques [14]. Therefore, measurements of MRD using NGS techniques are under development and not ready for routine application outside of clinical trials. 

In the near future, it is likely that ddPCR and NGS platforms, after standardization and validation of the results in prospective clinical trials, will be used for MRD detection [11].

### 3.2. NPM-Mutated AML

Ivey et al. used RT-PCR to detect MRD in 2569 samples from 346 patients with *NPM1*-mutated (*NPM1*m) AML who had undergone intensive treatment during the AML17 trial [16]. Of note, the presence of MRD, as determined by the quantitation of *NPM*1m transcripts, proved to be a powerful prognostic predictor, independent of other risk factors. Indeed, the persistence of *NPM1*m transcripts >0.01% in peripheral blood (PB) was associated with a greater risk of relapse at three years compared with the absence of such transcripts (82% vs. 30%; hazard ratio [HR], 4.80; 95% confidence intervals [CI], 2.95–7.80; *p* < 0.001) and a lower rate of survival (24% vs. 75%; HR, 4.38; 95% CI, 2.57–7.47; *p* < 0.001). The presence of MRD was the only independent prognostic factor for death at multivariate analysis (HR, 4.84; 95% CI, 2.57–9.15; *p* < 0.001). Overall, similar results were reached in another study in 229 adult patients enrolled in the Acute Leukemia French Association 0702 (ALFA-0702) trial [17]. Furthermore, after induction therapy, those patients who achieved a suboptimal reduction of *NPM1*m transcripts (<4-log) in the PB benefited from an allogeneic stem cell transplantation (ASCT) compared to those submitted to autologous SCT (AuSCT). The same benefit was not observed in patients achieving a significant reduction of MRD levels (>4-log).

Overall, these studies support the prognostic significance of *NPM1*m PB-MRD, independent of the cytogenetic and molecular context; *NPM1*m PB-MRD may also be used as a predictive factor for ASCT indication.

According to the first few clinical experiences in small *NPM1m* AML patient series, ddPCR demonstrated excellent sensitivity and agreement with RQ-PCR, also allowing for the detection of a variety of rare *NPM1* mutation subtypes [18,19]. These results suggest that ddPCR can effectively quantify *NPM1*m MRD, reducing the potential difficulties associated with NPM1 quantification, also in patients with unknown or rare mutant sequences.

### 3.3. Core-Binding Factor AML 

In 2012, Yin et al. assessed the clinical value of MRD monitoring in core-binding factor (CBF) AML by RT-qPCR in 278 patients enrolled in the United Kingdom MRC AML 15 trial [20]. Overall, they showed that rising MRD levels on serial monitoring were a strong predictor of relapse. These results were mirrored in other studies [21,22,23]. For instance, Williekens et al. showed that in t(8;21)(q22;q22) AML, MRD monitoring in PB every three months could predict hematological relapse and identify patients who could potentially benefit from therapy [21]. In a randomized study on 198 CBF-AML patients assigned to either a reinforced and a standard induction course followed by three high-dose cytarabine consolidation courses, cumulative incidence of relapse and relapse-free survival (RFS) at 36 months were 22% vs. 54% (*p* < 0.001) and 73% vs. 44% (*p* < 0.001) in patients who achieved 3-log MRD reduction versus the others [22].

#### 3.3.1. Acute Promyelocytic Leukemia

In the setting of acute promyelocytic leukemia (APL), Grimwade et al. used RT-PCR to detect leukemia-specific transcripts (e.g., PML–RARA, RARA–PML) in almost 7000 serial blood and marrow samples from 406 patients with newly diagnosed disease who were receiving all-trans-retinoic acid and anthracycline-based chemotherapy [24]. MRD monitoring was able to identify the majority of patients who were subject to relapse and was shown to be the most powerful predictor of RFS at multivariable analysis (HR, 17.87; 95% CI, 6.88–46.41; *p* < 0.0001). In particular, PCR negativity at the end of consolidation is associated with a low risk of relapse and a high chance of long-term survival.

#### 3.3.2. NOS AML and Other Categories

Schlenk et al. [25] showed that in FLT3-ITD AML, an allelic ratio (AR) ≥0.51 at diagnosis was associated with an unfavorable relapse-free survival (RFS) and OS. ASCT in first CR improved the outcome of patients with high AR, whereas no benefit was seen in patients with a low AR. Despite the clear prognostic impact of AR, at present, FLT3-ITD mutation is not regarded as a reliable MRD marker because it is not stable. In fact, FLT3-ITD-negative relapses are reported in approximately 25% of patients with a positive test at diagnosis. 

In a recent study of 193 adult patients with myeloid malignancies who underwent transplantation, RT-PCR WT-1 was used to estimate the presence of MRD [26]. Overall, standardized bone marrow levels using a 100-copy threshold in samples obtained before SCT at leukocyte recovery and during follow-up were able to provide relevant prognostic information. These results confirm those of a similar observation by Pozzi et al. Relapse was higher in 67 patients with WT-1 expression at any time post-ASCT, exceeding 100 copies (54%) compared with 16% for 55 patients with post-ASCT WT-1 expression <100 copies (*p* < 0.0001). Similarly, actuarial 5-year OS was 40% versus 63%, respectively (*p* = 0.03). In multivariate Cox analysis, WT-1 expression post-ASCT was the strongest predictor of relapse (HR, 4.5; *p* = 0.0001), independent of disease phase [27]. WT-1 transcript levels were also evaluated in leukapheresis (LK) used for AuSCT in 30 consecutive AML patients in CR and established a correlation with clinical outcome [28]. A cut-off level of 80 WT-1-LK copies/ABL 10^4^ copies to discriminate between positive and negative peripheral blood stem cell (PBSC) grafts, was strongly associated with disease recurrence, disease-free survival (DFS) and OS. However, it should be pointed out that, because of lack of specificity and limited sensitivity, clinical decisions based on WT-1 expression are recommended only in cases without any other MRD markers, including MFC. Indeed, WT-1 does not represent a leukemia-specific target, since it is also expressed by healthy bone marrow and hence presents low sensitivity. Therefore, WT-1 in PB is considered as more informative than when assessed in the bone marrow [29].

### 3.4. Flow Cytometry

The growing interest surrounding MFC is due to its wide applicability (>90% of AML), quickness, specificity, and ability to distinguish viable cells from bone marrow debris and dead cells. 

MRD monitoring by MFC relies on the expression on leukemic cells of a combination of antigens and/or flow cytometric physical abnormalities that are absent or very infrequent in normal bone marrow (e.g., cross-lineage expression, over-expression, reduced or absent expression, and asynchronous expression). Detection of leukemia-associated immunophenotypes (LAIP) or detection of different-from-normal (DfN) phenotypic patterns represent two complementary strategy of analysis [4]. The use of LAIP is based on the identification at diagnosis of immunophenotypically aberrant populations (a sort of patient’s “immunologic fingerprint”) that differ from normal hematopoietic cells; these immunological fingerprints are then used to trace residual leukemic cells after treatment. In the latter strategy of analysis, residual leukemic cells are identified as aberrant cell populations (i.e., LAIPs) within a normal pattern of differentiation by using a fixed antibody panel. Therefore, this strategy of analysis does not require the definition of an immunologic fingerprint at diagnosis. Differences between the LAIP and DfN approaches may be minimized if sufficiently large antibody panels (≥8 colors) are used for detection. Recent studies suggest that MRD evaluation by a ten-color panel together with the acquisition of a proper number of events improves the level of sensitivity of MFC assay and reduces the possibility to miss minor populations present at diagnosis that may eventually generate relapse [30,31,32]. In this regard, next-generation flow cytometry (NGF), which is a technique highly automated and based on the analysis of large numbers of cells (>10^7^ cells) with an optimized use of fluorochrome-conjugated antibody clones, will probably improve the power of outcome prediction of conventional eight- to ten-color MFC assay [14]. NGF has been recently reported to show significant utility in the monitoring of MRD in the setting of multiple myeloma [33] and acute lymphoblastic leukemia [34], suggesting that similar approaches may be exploited in AML.

One of the major concerns with MFC-MRD is that this technique requires considerable expertise and experience [4]; analysis and data interpretation may have some subjective elements and therefore potential biases, operator-dependent. Some of these problems can be reduced with standardized laboratory procedures including sample processing and instrument settings, single tube approaches with a pre-configured and stable assay, new automated interpretation software, central review, and continuous quality assessment [35,36,37,38]. In this view, scientific societies, such as European LeukemiaNet, are trying to endorse common approaches to define time-points, thresholds, panels, and results reporting of MFC-MRD [11]. 

Many studies have demonstrated that MRD detection by MFC provides strong prognostic information in AML after both induction and consolidation therapy. Which of these timepoints is optimal and the criterion for a positive test (e.g., >0% or >0.01% or >0.1%) is still unsettled (Table 2).

However, despite these issues, most of the studies identified two patient groups with either relatively poor or relatively good prognosis. MRD detection by MFC has clearly been shown to have a high positive predictive value for subsequent morphologic relapse, which usually occurs within 12 months of detection of MRD. In fact, several groups showed higher rate of relapse at one year in patients who tested MRD positive after two cycles of intensive chemotherapy [39,40,41]. The next step has been to combine post-treatment prognostic factors (evaluation of MRD status) with classical pre-treatment prognostic factors (cytogenetic and genetic finding), to better refine the prognosis of AML patients and, consequently, decide whether or not to intensify post-remission therapy with ASCT. In this way, two groups of patients, namely low-risk and high-risk patients, were identified and characterized with a different prognosis of a 4-year RFS and OS of 58% and 73% vs. 22% and 17%, respectively [42]. Cytogenetic/genetic findings at diagnosis and post-induction MFC analysis provided a robust means of stratifying patients also in the pediatric setting [43]. Despite these findings, however, there is not yet a general agreement on the role of MRD as a biomarker dictating the choice between ASCT and AuSCT/chemotherapy. A recent meta-analysis, including more than 1500 patients and 19 studies, tried to answer this issue and concluded that, overall, pre-transplant MRD positivity was associated with a worse leukemia-free survival (HR, 2.76 [1.90–4.00]), OS (HR, 2.36 [1.73–3.22], and cumulative incidence of relapse (HR, 3.65 [2.53–5.27], regardless of conditioning intensity, patient age, and detection method (MFC or RT-qPCR). Focusing only on MFC studies, however, a high degree of heterogeneity was observed, most likely due to site-specific methodological differences or differences in test performance and interpretation [12]. 

Although these results collectively suggest that pre-transplant MRD positivity should be considered as a negative predictive factor regardless of the therapeutic strategy, in these high-risk patients, the benefit of ASCT as compared with standard chemotherapy and/or AuSCT is relevant and evident. Therefore, currently, pre-transplant MRD positivity should not be considered a well-founded reason to hold back potentially curative ASCT. Further studies are needed to determine how MRD amount, should guide therapeutic decisions. 

### 3.5. Combination of Molecular Biology and Flow Cytometry

MFC and PCR measure two different things, namely malignant cells and mRNA expression in malignant cells. Currently, it is not possible to clearly establish which of these two techniques discriminates best between risk groups. Several studies evaluated tandem MRD analyses by MFC and RT-qPCR in AML. In a pilot study, Rossi et al. evaluated MRD in 30 adult AML patients by MFC and WT-1 expression before and after ASCT [51]. Overall, diagnostic performance of pre-transplant MRD measured by MFC was higher than that obtained by WT-1 expression; similar results were displayed at 30 days post-transplant, while better values by WT-1 compared with MFC were reported at day 90. Inaba et al. evaluated samples from 203 children and adolescents with newly diagnosed AML enrolled in the AML02 study [7]. Virtually all (308/311; 99.0%) MRD-negative samples by PCR were also MRD negative by MFC. However, only 19 (9.6%) of the 197 PCR-positive samples were positive at MFC, with the analysis of AML1-ETO and CBFbeta-MYH11 accounting for most discrepancies. Moreover, MRD by MFC after induction was a predictor of lower event-free survival and higher relapse rate; prediction was not improved by morphologic information or molecular findings. A more recent study evaluated 42 patients with t(8;21)(q22;q22)/RUNX1-RUNX1T1 and 51 with inv(16)(p13.1q22)/CBFBMYH11 [52]. Overall, the agreement between MFC and RT-qPCR was weak, and the best correlation was found for very low (<0.1%) or high level (≥10%) of fusion transcripts detected by RT-qPCR. The post-induction bone marrow MFC study was useful in predicting AML relapse, particularly in patients with discordant MFC and RT-qPCR results. On the contrary, during clinical follow-up, MFC results were less sensitive compared with RT-qPCR to detect residual leukemic cells that predicted early relapse. Therefore, taken together, these data suggest that the two techniques provide complementary information for MRD assessment and can be used in tandem for “universal” monitoring of MRD in patients with CBF AML. Similarly, the combination of molecular- and MFC-based MRD assessment may improve the prognostic value of pre-transplant MRD evaluation and might be useful in the selection of the intensity of conditioning [53]. More recently, NGS has been increasingly applied to assess post-treatment persistence of various mutations. A recent study of 340 adult AML patients demonstrated the feasibility of combining MFC and NGS for the purpose of MRD monitoring, reporting concordant findings between both methods in 69% of cases. In the discordant cases, NGS was positive in approximately 60% and MFC in approximately 40%. Combining NGS and MFC appeared to improve the sensitivity/predictive value of a positive MRD test [54]. Similar results have been reported for prediction of post-ASCT relapse [31]. In addition, Morita et al. reported that post-treatment clearance of diagnostic mutations might help to better stratify MFC-negative patients for the risk of relapse [55].

## 4. Use of MRD to Evaluate Efficacy of New Drugs 

Currently, survival is most frequently used as the endpoint to demonstrate clinical benefit of novel drugs and/or novel drug combinations. Limitations of using survival as an endpoint for MRD include the long duration of follow-up and the presence of confounding factors, such as post-remission or rescue therapies, which may be heterogeneously applied to different patients. 

These secondary interventions may range from support and palliation to standard chemotherapy and/or enrollment into experimental drug trials, with or without ASCT, with unpredictable results in terms of disease-free intervals subsequent to first CR. Using biomarkers (i.e., MRD) as an endpoint, has the potential to more quickly demonstrate a benefit (or lack thereof) of a given treatment, compared with survival by itself, ideally by studying the predictive power of MRD for CR duration. For instance, it has been recently shown that testing of MRD may represent a tool to drive treatment de-escalation in patients who achieve an MRD-negative CR, especially during their first cycle of therapy [56]. Moreover, they can be used to identify patients who are most suitable for a given treatment. This may also have implications when designing a clinical trial, since it is plausible that response rates to new drugs could be higher if tested when MRD is the only evidence of disease [57]. Moreover, patients who are not likely to benefit from a given therapy may be excluded and premature interruption of a study can be conceived if patients do not achieve benefits, making it easier for the authorization of new molecules [58,59].

To date, several studies have been conducted to assess the potential of new therapies in overcoming MRD [41,60,61,62,63,64,65]. Ragon et al. have investigated whether maintenance therapy with hypomethylating agents (HMA), including decitabine and azacitidine after induction/consolidation, can be used for MRD elimination and prolonging RFS [60]. A total of 23 patients with CBF-AML that received HMA therapy following induction/consolidation with fludarabine, cytarabine, and G-CSF (FLAG) with low-dose gemtuzumab or idarubicin were evaluated by RT-PCR. Although the low number of patients enrolled hampers the analysis, this study suggested that CBF-AML patients with low levels of RT-PCR (0.01–0.05) at the end of induction/consolidation chemotherapy might have some benefit from maintenance HMA, particularly those that have a reduction in the RT-PCR within the first two cycles of HMA therapy. In a small study (*n* = 59) Platzbecker et al. evaluated azacitidine as treatment of MRD in patients with either CD34^+^ myelodysplastic syndromes or AML after HSCT, showing that this molecule might prevent or delay hematologic relapse [63]. Moreover, at a median of 169 days after HSCT, 20 out of 59 patients experienced a decrease of CD34^+^ donor chimerism to <80% and received four azacytidine cycles (75 mg/m^2^/day for 7 days) during the remission period. A total of 16 patients (80%) responded, showing either increasing CD34^+^ donor chimerism to ≥80% (*n* = 10; 50%) or stabilization (*n* = 6; 30%) in the absence of relapse. 

Gemtuzumab ozogamicin (GO) is active for the treatment of CD33^+^ AML and may improve the outcome of specific patient subgroups in combination with conventional chemotherapy. A small study on pediatric patients showed that both GO alone (*n* = 17) and GO + chemotherapy (*n* = 29), may reduce MRD before HSCT and was not found to be associated with increased treatment-related mortality after transplantation [64]; overall, similar results were reported in a larger (*n* = 130) prospective trial on GO and FLAI in adult patients [66]. In this latter study, after induction with FLAI-GO, CR rate was 82%, with a manageable toxicity: only 45% of patients experienced transient and reversible adverse events related to treatment. The rates of 1-, 2- and 5-year OS were 80%, 63%, and 52%, respectively. However, in another larger randomized study in 183 patients with WT-1 overexpression and in 77 patients with *NMP1*m at diagnosis, the achievement of a negative *NPM1* MRD was surprisingly more frequent in patients treated with GO compared with those assigned to the control arm after induction (39% vs. 7%; *p* = 0.006) and at the end of treatment (91% vs. 61%; *p* = 0.028) [61]. 

Midostaurin is a prototype kinase inhibitor, originally developed as a protein kinase C inhibitor and subsequently as an angiogenesis inhibitor given its ability to inhibit vascular endothelial growth factor (VEGFR) [67]. Years later, midostaurin was shown to be a potent inhibitor of the FLT3 tyrosine kinase and to have activity against mutant forms of KIT receptor tyrosine kinase which drive advanced systemic mastocytosis (SM). Midostaurin in combination with standard chemotherapy was also evaluated in the Cancer and Leukemia Group B 10603/RATIFY study, a large, phase III, randomized, placebo-controlled trial in patients with newly diagnosed *FLT3*-mutated AML [68]. This was the first study to show significant and clinically relevant improvements in OS and event-free survival with the addition of a targeted therapy to standard chemotherapy in this population, although the response rate was similar in the two groups. These data allow us to speculate that midostaurin can influence MRD and lead to improved quality of response and represent the basis for a planned study evaluating MRD in AML patients with FLT3 expression treated with midostaurin + chemotherapy.

## 5. Conclusions

The evaluation of MRD should be considered a major tool to assess the efficacy of chemotherapy or targeted therapy in AML patients, beyond morphological parameters. Owing to other new, highly subset-specific and variably effective drugs being tested and introduced, such as the recently FDA-approved IDH1 and IDH2 inhibitors [69] and the bcl2 inhibitor venetoclax [70], there is an increased need of accurate MRD monitoring. Consequently, this would allow us to use relatively well tolerated agents very early at the time of molecular resistance or relapse (i.e., prior to morphologic relapse, at which point the patients are frequently symptomatic and less likely to respond). Indeed, a proper assessment of MRD using dedicated techniques allows a precise estimation of the quality of response to therapy. Therefore, clinical studies in AML patients do need to include the evaluation of MRD as a major endpoint. 

## Figures and Tables

**Table 1:** Response criteria in acute myeloid leukemia (AML) (modified from Dohner 2017).

Category	Definition
**Response**	
CR_MRD-_	If MRD marker identified pretreatment, CR with negativity for a genetic marker by RT-qPCR, or CR with negativity by MFC
CR	Bone marrow blasts <5%; absence of circulating blasts and blasts with Auer rods; absence of extramedullary disease; ANC ≥10 × 10^9^/L; platelet count ≥100 × 10^9^/L (100,000/µL); independency from blood transfusions
CR_i_	All CR criteria except for incomplete blood count recovery with residual neutropenia (<10 × 10^9^/L [1000/µL]) or thrombocytopenia (<100 × 10^9^/L [100,000/µL])
MLFS	Bone marrow blasts <5%; absence of blasts with Auer rods; absence of extramedullary disease; no hematologic recovery required
PR	All hematologic criteria of CR; decrease of bone marrow blast percentage to 5–25%; and decrease of pretreatment bone marrow blast percentage by at least 50%
**Treatment failure**	
Primary refractory disease	No CR or CR_i_ after two courses of intensive induction treatment; excluding patients with death in aplasia or death due to an indeterminate cause
Death in aplasia	Deaths occurring ≥7 days following completion of initial treatment while cytopenic, with an aplastic or hypoplastic bone marrow obtained within 7 days of death, without evidence of persistent leukemia
Death from indeterminate cause	Deaths occurring before completion of therapy, or <7 days following its completion; or deaths occurring ≥7 days following completion of initial therapy with no blasts in the blood, but no bone marrow examination available
**Response criteria for clinical trials online**	
Stable disease	Absence of CR_MRD_, CR, CR_1_, PR, MLFS; and criteria for PD not met
PD *^,^†	Evidence for an increase in bone marrow blast percentage and/or increase of absolute blast counts in the blood: >50% increase in marrow blasts over baseline (a minimum 15% point increase is required in cases with <30% blasts at baseline; or persistent marrow blast percentage of >70% over at least 3 months; without at least a 100% improvement in ANC to an absolute level >0.5 × 10^9^/L (500/µL), and/or platelet counts to >50 × 10^9^/L (50,000/µL) nontransfused; or >50% increase in peripheral blasts (WBC × % blasts) to >25 × 10^9^/L (>25,000)/µL) in the absence of differentiation syndrome ^†^; or Newly extramedullary disease
**Relapse**	
Hematologic relapse (after CR_MRD_, CR, CR_i_)	Bone marrow blasts ≥5%; or reappearance of blasts in the blood; or development of extramedullary disease
Molecular relapse (after CR_MRD-_)	If MRD marker identified pretreatment, reoccurrence of MRD as assessed by RT-qPCR or by MFC

ANC: absolute neutrophil count; CR: Complete remission; CR_MRD-_: CR without minimal residual disease; CR_i_: CR with incomplete hematologic recovery; MLFS: Morphologic leukemia-free state; PR: partial remission; PD: progressive disease; RT-qPCR: real-time quantitative polymerase chain reaction; MFC: multiparameter flow cytometry; WBC: white blood cell. * The authors acknowledge that this new provisional category is arbitrarily defined; the category aims at harmonizing the various definitions used in difference clinical trials. ^†^ Certain targeted therapies, for example, those inhibiting mutant isocitrate dehydrogenase (IDH) proteins, may cause a differentiation syndrome, that is, a transient increase in the percentage of bone marrow blasts and an absolute increase in blood blasts; in the setting of therapy with such compounds, an increase in blasts may not necessarily indicate PD.

**Table 2:** Available studies on MRD detection by MFC provides strong prognostic information in AML after both induction and consolidation therapy.

Study	Patients (*n*)	Timepoint	Multivariate Analysis	Threshold Post-IND	Threshold Post-CONS	Method	Details on Survival Parameters
[44]	53	I, C	I, C	0.5%	0.2%	NA	
[45]	56	I, C	C	0.45%	0.035%	Empirical	
[46]	126	I	I	<0.01%,	NA	NA	MRD > 1%: 3-year RR: 85%
				0.01–0.1%,			MRD 0.1–1.0%: 3-year RR: 45%
				0.1–1%,			MRD 0.01–0.1%: 3-year RR: 14%
				>1%			MRD <0.01%: 3-year RR: 0%
[47]	62	I, C	C	Continuous analysis log-difference	Continuous analysis log-difference	75th percentiles of log-difference	
[48]	106	Day 16 after I	Day 16 after I	Continuous analysis log-difference	NA	Median of log-difference	
[49]	100	I, C	C	0.035%	0.035%	Maximally selected log-rank statistics	5-year RFS 72% (MRD neg) vs. 11% (MRD pos)
[39]	142	I, C	C	0.035%	0.035%	Maximally selected log-rank statistics	5-year RFS 60% (MRD neg) vs. 16% (MRD pos)
[50]	54	I	I	0.15%	0.15%	ROC analysis	
[40]	241	I 1, I 2, C	I 2	0.1%	0.1%	NA	Cutoff points between 0.05 and 0.8 are all significant

I: induction; C: Consolidation; NA: not available; MRD: minimal residual disease; RR: risk of relapse; RFS: relapse free survival; ROC: receiver operating characteristics.
